# A Case of Filter Retrieval With an Aspiration Catheter for Filter Occlusion in Emergency Carotid Artery Stenting

**DOI:** 10.7759/cureus.76684

**Published:** 2024-12-31

**Authors:** Yuka Ogawa, Yoichi Morofuji, Yohei Tateishi, Takayuki Matsuo

**Affiliations:** 1 Department of Neurosurgery, Nagasaki University Graduate School of Medicine, Nagasaki, JPN; 2 Department of Neurosurgery, Nagasaki University, Nagasaki, JPN; 3 Department of Neurology and Strokology, Nagasaki University, Nagasaki, JPN

**Keywords:** aspiration catheter, carotid artery stenting, embolic protection device, extension wire, internal carotid artery occlusion

## Abstract

During emergency carotid artery stenting (CAS), the obstruction of filters utilized for safeguarding against distal embolization, sometimes caused by substantial plaque or thrombus accumulation, is a frequent challenge. We present the case of an 81-year-old man who underwent emergency CAS in which the filter became occluded and was successfully retrieved using an aspiration catheter, leading to a favorable clinical outcome. Employing an aspiration catheter for filter retrieval is an option for restoring anterograde blood flow and mitigating the risk of distal embolization.

## Introduction

The management of acute symptomatic extracranial internal carotid artery occlusions (ICAOs) remains individualized because of the lack of randomized controlled trial (RCT) data [1]. In emergency carotid artery stenting (CAS), occlusion of filters used for distal protection due to large amounts of plaque is often a problem [2,3]. Herein, we report a case of filter obstruction in which the filter was retrieved using an aspiration catheter.

## Case presentation

**Figure 1:**
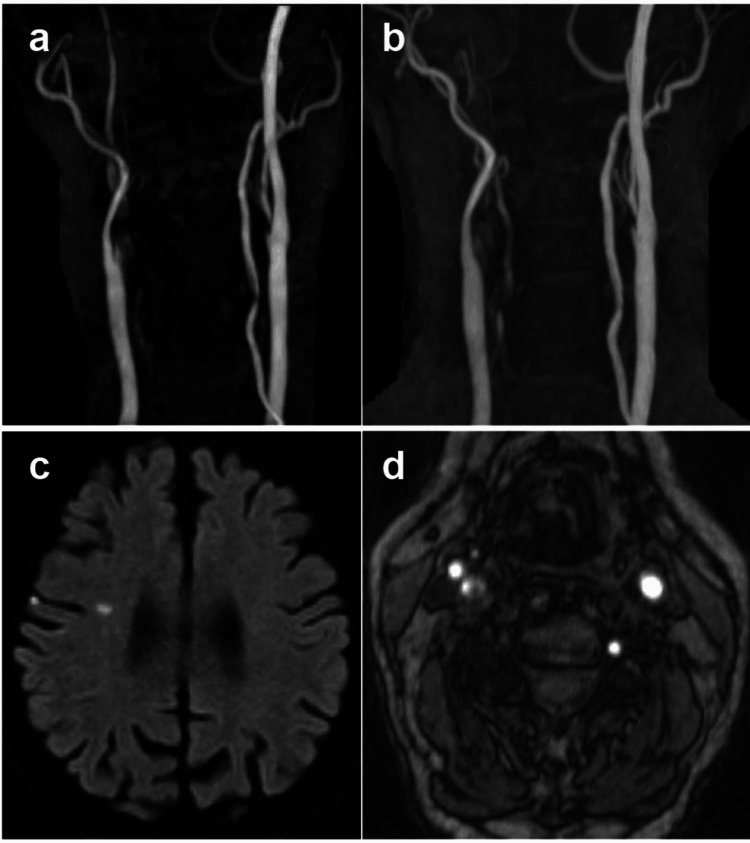
MRA and diffusion-weighted images on admission. (a) MRA performed one month previously showed severe stenosis of the right ICA. (b) MRA on admission revealed occlusion of the right ICA at the cervical portion. (c) Diffusion-weighted images revealed scattered, small, high-intensity lesions in the right frontal lobe cortex. (d) TOF images showed high-intensity signals in plaque MRA: magnetic resonance angiography; TOF: time of flight

An 81-year-old man presented with repeated recurrent left-hand weakness within 24 hours after one-week clopidogrel discontinuation due to total cystectomy. He had a clinical history of laryngeal cancer that had been treated with radiation therapy five years previously. The patient was diagnosed with progressive asymptomatic right internal carotid artery (ICA) stenosis, probably due to radiation. Diffusion-weighted images revealed scattered, small, high-intensity lesions in the right frontal lobe cortex. MR angiography (MRA) revealed occlusion of the right ICA at the cervical portion. Ultrasonography and MRA performed one month previously showed severe stenosis of the right ICA (Figure 1). Considering his clinical course and magnetic resonance imaging (MRI) findings, the right ICA occlusion may have occurred within 24 hours. We planned to perform a diagnostic angiography and CAS.

**Figure 2:**
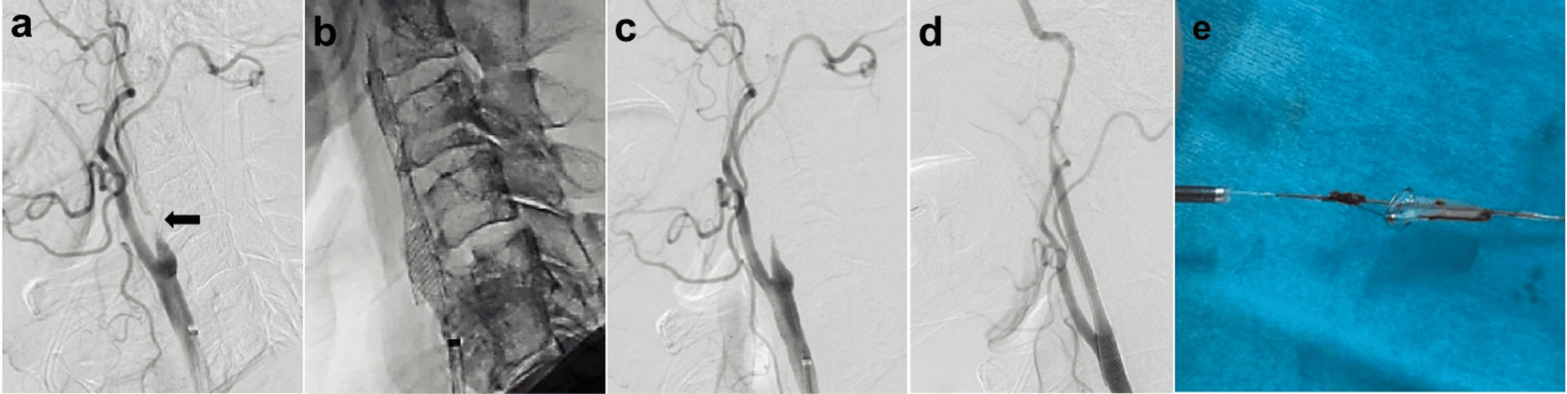
Procedure of carotid artery stenting. (a) Initial common carotid angiogram showed carotid near-occlusion (arrow). (b) Carotid Wallstent was delivered to the stenotic lesion and deployed after filter protection. (c) After stent deployment, carotid angiography showed flow stagnation in the ICA. (d) Final common carotid angiography showed that the stenosis was well dilated, and anterograde blood flow was fully restored after successfully retrieving the FilterWire EZ with an aspiration catheter. (e) A large red thrombus adhering to the retrieved filter device was observed ICA: internal carotid artery

All procedures were performed under local anesthesia. The patient received dual antiplatelet therapy with a loading dose before the procedure. A 9-Fr balloon guide catheter (Optimo, Tokai Medical Products, Aichi, Japan) was navigated into the right common carotid artery using the transfemoral approach. Right common carotid angiography revealed near-occlusion (Figure 2a). After inflation of the balloon guide catheter, a FilterWire EZ (Boston Scientific, Natick, MA) was delivered to the distal end of the stenotic lesion for distal protection. After predilatation with a 3.0- × 40-mm Coyote angioplasty balloon (Boston Scientific, Marlborough, MA), an 8.0-mm self-expanding Carotid WALLSTENT (Boston Scientific, Santa Clara, CA) was delivered to the stenotic lesion and deployed (Figure 2b). After stent deployment, carotid angiography showed flow stagnation in the distal to the ICA stent (Figure 2c). Regardless of repeated postdilatation with a 4.5- x 30-mm Sterling angioplasty balloon (Boston Scientific, Marlborough, MA), the flow stagnation did not change. This was expected to be due to the plugging of the filter by a large amount of atherosclerotic plaque. We decided to use an aspiration catheter, which has a larger diameter, instead of the usual capture sheath because we suspected that the capture sheath was not sufficient to withdraw the high-volume plaque and that there was a high risk of distal embolization. To use an aspiration catheter to retrieve FilterWire EZ, we needed to connect the end of the Filterwire EZ with an extension wire (Asahi Intecc, Aichi, Japan) to obtain sufficient length to deliver an aspiration catheter. We successfully retrieved the FilterWire EZ using a REACT 71 aspiration catheter (Medtronic Neurovascular, Irvine, CA) under proximal control using an Optimo balloon guide catheter. We led REACT proximal to the filter, and continuous aspirations were performed from the Optimo and REACT to capture the filter in REACT. Right common carotid angiography showed that the stenosis was well dilated, and anterograde blood flow was fully restored (Figure 2d). A large thrombus adhering to the retrieved filter device was observed (Figure 2e), which was considered to be the cause of stagnation of blood flow. Pathological examination revealed a white thrombus composed mainly of fibrin and inflammatory cell infiltrates. No atheromatous components, such as cholesterin crystals or foam cells, were detected.

**Figure 3:**
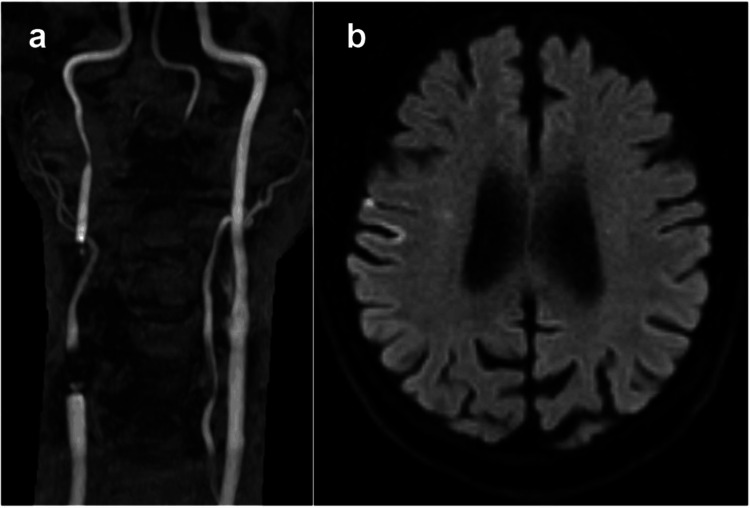
Postoperative MRA and diffusion-weighted images. (a) Postoperative MRA indicated an improvement in the occlusion of the right ICA. (b) Diffusion-weighted images showed no new ischemic lesions MRA: magnetic resonance angiography; ICA: internal carotid artery

The patient tolerated the procedure without any complications. Postprocedural MRI showed no new ischemic lesions (Figure 3). His transit ischemic attack symptoms were completely resolved, and he was discharged home. At six-month follow-up, MRA and carotid ultrasound revealed no recurrence of carotid artery stenosis. His modified Rankin scale at six months was 0.

This article was previously presented as a meeting abstract at the 11th Japan-Korea Joint Stroke Conference on November 16, 2023.

## Discussion

Treatment for acute and symptomatic extracranial ICAOs remains individualized and highly dependent on expert opinions owing to the lack of randomized controlled data. At the very least, near-occlusion with full collapse (distal ICA < 2.0 mm and/or ICA ratio < 0.42) has been reported to carry a high risk of early stroke recurrence [4], and it is reasonable to perform more aggressive treatment in such cases. Intravenous thrombolysis is beneficial to patients with acute ischemic stroke (AIS). However, the low recanalization rate and incidence of symptomatic intracranial hemorrhage are problematic [1]. Mechanical thrombectomy (MT) is an effective treatment for AIS resulting from large vessel occlusion; however, RCTs of MT have not included patients with extracranial ICAOs. However, in nonrandomized trials, endovascular therapy, including MT, percutaneous transluminal angioplasty, and CAS, has been suggested for its safety and efficacy [5]. In the presence of clinical-imaging perfusion mismatches and hemodynamic instability, patients with acute extracranial ICAOs can experience worsening or recurrence of symptoms, so they should be predicted early and treated aggressively [6].

Radiotherapy to the neck often causes carotid artery stenosis. It has been shown by cross-sectional and retrospective studies that patients are at risk of signiﬁcant carotid stenosis, and a high proportion (17%) are symptomatic [7]. Radiation-induced carotid artery injury is attributed to mechanisms such as vessel inflammation, carotid atherosclerosis, intimal proliferation, medial necrosis, and peri-intimal fibrosis [8]. Of these, atherosclerosis is said to be the most important factor in radiation-induced stenosis. Radiation directly damages endothelial cells, causing changes in endothelial function and increased permeability, which provides the conditions for platelet adhesion and mononuclear cell migration into the arterial wall. This results in the proliferation of arterial smooth muscle cells and the maintenance of chronic inflammation, leading to accelerated atherosclerosis [9]. One clinical research reported that radiotherapy causes more rapid progression of carotid artery stenosis compared with nonirradiated atherosclerotic arteries [7].

In the present case of radiation-induced ICA stenosis, clopidogrel discontinuation for total cystectomy led to the worsening of vascular endothelial damage in the stenotic region and induced a platelet thrombus. Due to the large number of platelet thrombi, acute and symptomatic ICA occlusion occurred. Although the time of onset was unclear, an emergency CAS was performed. Owing to the large amount of thrombus, occlusion of the filters used for distal protection occurred. It was considered that not plaque contents but a large amount of adherent platelet thrombus occluded the filter. The filter was retrieved with an aspiration catheter, and anterograde blood flow was recovered.

During CAS procedures, embolus protection devices are commonly used because of concerns regarding distal embolization. Hayashi et al. [2] reported that flow impairment during CAS with an embolus protection filter was observed in 10 cases (19.2%). They reported that it was induced by a filter obstruction or vasospasm. In all cases, the flow was restored after filtering.

Whether to use a capture sheath or aspiration catheter to retrieve the filter is an expert opinion. The efficacy of filter retrieval using an aspiration catheter, as in the present case, has been reported by Han et al. [3]. It is thought to reduce the spreading of debris during filter retrieval because it has a wider diameter than the capture sheath. In cases where high plaque or thrombus volumes are expected, using an aspiration catheter instead of a capture sheath is an option to reduce the risk of distal embolization. Delivering an aspiration catheter using an extension wire of sufficient length is possible.

## Conclusions

In cases of emergency CAS, occlusion of the embolus protection filters and stagnation of blood flow, as in this case, can occur. Anterograde blood flow must be restored immediately and the spreading of debris should be minimized during filter retrieval. It appears that the method of retrieving the filter using an aspiration catheter with a wider diameter is effective.

## References

[1] Romoli M, Mosconi MG, Pierini P (2021). Reperfusion strategies in stroke due to isolated cervical internal carotid artery occlusion: systematic review and treatment comparison. Neurol Sci.

[2] Hayashi K, Horie N, Morikawa M (2014). Pathophysiology of flow impairment during carotid artery stenting with an embolus protection filter. Acta Neurochir (Wien).

[3] Han SH, Kang WC, Ahn TH, Shin EK (2009). Pseudo-no-reflow phenomenon in carotid artery stenting using FilterWire EX: successful recovery by aspiration thrombectomy. J Korean Med Sci.

[4] Henze A, Fox AJ, Johansson E (2024). High risk of early recurrent stroke in patients with near-occlusion with full collapse of the internal carotid artery. Neuroradiology.

[5] Kargiotis O, Psychogios K, Safouris A (2022). Diagnosis and treatment of acute isolated proximal internal carotid artery occlusions: a narrative review. Ther Adv Neurol Disord.

[6] Jadhav A, Panczykowski D, Jumaa M (2018). Angioplasty and stenting for symptomatic extracranial non-tandem internal carotid artery occlusion. J Neurointerv Surg.

[7] Cheng SW, Ting AC, Ho P, Wu LL (2004). Accelerated progression of carotid stenosis in patients with previous external neck irradiation. J Vasc Surg.

[8] Zheng Z, Zhao Q, Wei J (2020). Medical prevention and treatment of radiation-induced carotid injury. Biomed Pharmacother.

[9] Ramadan R, Vromans E, Anang DC (2019). Single and fractionated ionizing radiation induce alterations in endothelial connexin expression and channel function. Sci Rep.

